# Preparation and Characterization of Composites Based on ABS Modified with Polysiloxane Derivatives

**DOI:** 10.3390/ma17030561

**Published:** 2024-01-24

**Authors:** Bogna Sztorch, Roksana Konieczna, Daria Pakuła, Miłosz Frydrych, Bogdan Marciniec, Robert E. Przekop

**Affiliations:** 1 Centre for Advanced Technologies, Adam Mickiewicz University Poznan, Uniwersytetu Poznańskiego 10, 61-614 Poznan, Poland; rokkon@amu.edu.pl (R.K.); darpak@amu.edu.pl (D.P.); frydrych@amu.edu.pl (M.F.); bogdan.marciniec@amu.edu.pl (B.M.); rprzekop@amu.edu.pl (R.E.P.); 2 Faculty of Chemistry, Adam Mickiewicz University in Poznań, Uniwersytetu Poznańskiego 8, 61-614 Poznan, Poland

**Keywords:** ABS, FDM, polysiloxane, silica, 3D printing

## Abstract

In this study, organosilicon compounds were used as modifiers of filaments constituting building materials for 3D printing technology. Polymethylhydrosiloxane underwent a hydrosilylation reaction with styrene, octadecene, and vinyltrimethoxysilane to produce new di- or tri-functional derivatives with varying ratios of olefins. These compounds were then mixed with silica and incorporated into the ABS matrix using standard processing methods. The resulting systems exhibited changes in their physicochemical and mechanical characteristics. Several of the obtained composites (e.g., modified with VT:6STYR) had an increase in the contact angle of over 20° resulting in a hydrophobic surface. The addition of modifiers also prevented a decrease in rheological parameters regardless of the amount of filler added. In addition, comprehensive tests of the thermal decomposition of the obtained composites were performed and an attempt was made to precisely characterize the decomposition of ABS using FT-IR and optical microscopy, which allowed us to determine the impact of individual groups on the thermal stability of the system.

## 1. Introduction

Additive technologies, commonly known as 3D printing, are an intensively developing field that involves the use of a three-dimensional virtual model to create a real object layer-by-layer. During printing, materials such as polymers, ceramics, or metal can be bonded together permanently when exposed to a desired temperature or a laser beam. The rapid development of additive technologies began in 2009 when most of the patents for 3D printing devices expired, which made it possible to use the technology on a wider scale [1].

Three-dimensional printing currently offers a wide spectrum of applications. It is used in the construction [2,3], automotive [4,5], medical [6,7,8], decoration, machinery, dental, and textile industries. Among the most important advantages of additive technologies is the ability to produce models with complex geometry without the need for multiple manufacturing tools. Disadvantages include limited dimensional accuracy, the problem of printing skewed surfaces, and the frequent need for additional surface treatments. Compared with conventional methods (i.e., milling and turning), additive techniques are a relatively young and rapidly growing discipline. Therefore, their disadvantages are gradually being eliminated through the introduction of design improvements and the development of a new range of materials suitable for 3D printing [9].

FDM (*Fused Deposition Modeling*) is one of the most commonly used incremental techniques. It creates three-dimensional objects by adding successive layers of molten material. The printout is based on the generated digital 3D object and provides us with the ability to manipulate the geometry of an object. The possibility of obtaining models with a complex geometry combined with a shorter design time and lower production costs make this technique very popular [10,11].

Thermoplastics are attractive materials for use in FDM as is the older technology of injection molding. Polymers such as acrylonitrile butadiene styrene (ABS), polylactide (PLA), and polyamide are among the best ones available [12]. ABS has been used in the electronics sector (e.g., for use in the bodies of electrical devices), sports, the automotive [4,5,13], household appliances (e.g., for use in the bodies of TV sets and radio receivers), and toys [14,15,16]. This material is characterized by its hardness and good mechanical strength, which have also been observed at low temperatures. In addition, ABS does not conduct electricity, has high impact resistance, and is resistant to high temperatures [17]. It owes its chemical resistance and thermal stability to the presence of acrylonitrile units in the polymer chain. Aromatic groups affects the stiffness and processability of the material. The butadiene phase, on the other hand, is responsible for improved impact strength and hardness [18]. One of the main disadvantages of using this material is its low adhesion to the surface during printing, and deformations occurring during its shape formation are another disadvantage [19]. Numerous publications have aimed to mitigate these effects through material modifications through the addition of different fillers while simultaneously enhancing mechanical or processing characteristics [20,21,22,23,24]. In our work, we presented the preparation of ABS composites with a modified silica nanofiller. There are well-known applications of nanofillers in the literature; Kim, I.-J. et al. [25] in their work described ABS nanocomposites with silica nanoparticles obtained through emulsion polymerization techniques and compress molding in their research. The composites produced through this method exhibited a noteworthy boost in impact strength, with an approximate enhancement of 30%.

In work [26] the influence of nanoparticles, i.e., montmorillonite, CaCO_3_, silica, and multiwalled carbon nanotubes, on the mechanical properties of ABS composites produced using the FDM technique was examined. It was found that the addition of fillers improved the mechanical strength and thermal stability of the samples. Moreover, some of the obtained composites were characterized by increased bending strength and reduced mechanical anisotropy.

In the work of Bai Huang et al. [27], silica-based modifiers were also produced to modify ABS for 3D printing using FDM technology. They optimized the efficiency of 3D printing acrylonitrile–butadiene–styrene composites through cellulose nanocrystal/silica nanohybrids (CSNs), resulting in composites with increased adhesion between layers. CSNs obtained through the TEOS sol–gel method have uniformly distributed nanosilica on their surfaces, and nanohybrids demonstrated both an excellent efficiency and well-reinforcement effect in FDM. In our previous work, we thoroughly discussed the impact of functionalized organosilicon additives in the context of other thermoplastics such as polyethylene (PE) and polylactide acid (PLA) [28,29].

This paper presents the surface modification of silica using organosilicon compounds (polysiloxane derivatives) and then introduces the filler into the polymer matrix. The obtained composites were tested for processing properties (through the mass flow index) and physicochemical properties (through surface analysis and mechanical properties). Microscopic images (through an optical microscope) were taken to determine the morphology and dispersion of the filler in the matrix. Differential scanning calorimetry (DSC) and thermogravimetric analysis (TG) were also performed to determine thermal properties. The investigation conducted here facilitates the assessment of the impact of modified nanosilica on the resultant composites and also enables the evaluation of the potential applications of the material obtained.

## 2. Materials and Methods

### 2.1. Materials

ABS type TR558 was purchased from LG Chem (Seul, South Korea), Fumed silica filler AEROSIL^®^ 200 (Aero) with a specific surface area of 200 m^2^/g was purchased from Evonik (Essen, Germany)

The chemicals were purchased from the following sources:

Polymethylhydrosiloxane, trimethylsilyl-terminated, 15–25 cSt from Gelest (Morrisville, PA, USA); styrene (STYR), octadecene (OD), toluene, chloroform-d, Karstedt’s catalyst xylene solution from Merck KGaA (Darmstadt, Germany); vinyltrimethoxysilane (VT) from BRB; and P_2_O_5_ from Avantor Performance Materials Poland S.A. (Gliwice, Poland)
Toluene was degassed and dried by distilling it from P_2_O_5_ under an argon atmosphere.

### 2.2. Analyses

Fourier transform-infrared (FT-IR) spectra were recorded on a Nicolet iS 50 Fourier transform spectrophotometer (Thermo Fischer Scientific, Waltham, MA, USA) equipped with a diamond ATR unit with a resolution of 0.09 cm^−1^.

^1^H, ^13^C, and ^29^Si nuclear magnetic resonance (NMR) spectra were recorded at 25 °C on Bruker Ascend 400 and Ultra Shield 300 spectrometers using CDCl_3_ as a solvent. Chemical shifts were reported in ppm concerning the residual solvent (CHCl_3_) peaks for ^1^H and ^13^C.

The melt flow rate (MFR) was measured using the Instron CEAST MF20 melt flow tester according to the standard [30] at 220 °C for the load of 5 kg, and the time of cutting off the polymer stream was 30 s.

Water contact angle (WCA) analysis were performed through the sessile drop technique at room temperature and atmospheric pressure using a Krüss DSA100 goniometer. Three independent measurements were taken for each sample, each with a 5 µL water drop, and the obtained results were averaged.

Light microscopy images of the surface and fractures of the composites were taken using a KEYENCE VHX-7000 digital microscope (Keyence International, Mechelen, Belgium, NV/SA) with a 100–1000 VH-Z100T zoom lens. All images were recorded using a VHX 7020 camera.

Tensile and flexural strength tests were performed using the universal testing machine INSTRON 5969 with a maximum measuring capability of 50 kN. Seven samples were selected from each system, placed in the testing machine, and subjected to tensile and flexural tests. For each modifier, seven values of stress, modulus of elasticity, and elongation were obtained, which were then averaged. The traverse speed for the tensile strength measurements was set at 2 mm/min.

A Charpy impact test (with no notch) was performed on an Instron Ceast 9050 impact machine according to the [31] standard. For all the series, 6 measurements were performed for each material. 

Hardness of the composite samples was tested through the Shore method using a durometer from Bareiss Prüfgerätebau GmbH (Oberdischingen, Germany).

Thermogravimetry (TGA) was performed using a NETZSCH 209 F1 Libra gravimetric analyzer (Selb, Germany). Samples of 9 ± 0.5 mg were cut from each granulate and placed in Al_2_O_3_ crucibles. Measurements were conducted under nitrogen and air (flow of 20 mL/min) within various temperature ranges, i.e., from 30 °C to 390 °C, from 30 °C to 400 °C, from 30 °C to 455 °C, or from 30 °C to 500 °C and a 10 °C/min heating rate. 

Differential scanning calorimetry (DSC) was performed using a NETZSCH204 F1 Phoenix calorimeter. Samples of 6 ± 0.2 mg were placed in an aluminum crucible with a punctured lid. The measurements were performed under nitrogen within a temperature range of −20 °C to 310 °C and at a 10 °C/min heating rate.

### 2.3. The Procedure for Synthesis of Polysiloxane Derivatives

In a typical procedure, a 500 mL three-neck round bottom flask was charged with 30 g of polymethylhydrosiloxane, 250 mL of toluene, and calculated amounts of olefins (Table 1). The reaction mixture was set at 70 °C and, before reaching boiling point, Karstedt’s catalyst (10^−5^ eq Pt/mol SiH) solution was added, which resulted in a quick increase in temperature and the system starting to reflux. The reaction mixture was kept at reflux and samples were taken for FT-IR control until full Si–H group disappearance was observed. Then, the solvent was evaporated to dryness under a vacuum to obtain a pure analytical sample.

### 2.4. The Procedure of Mixing Nanosilicate with a Modifier

**Figure 1:**
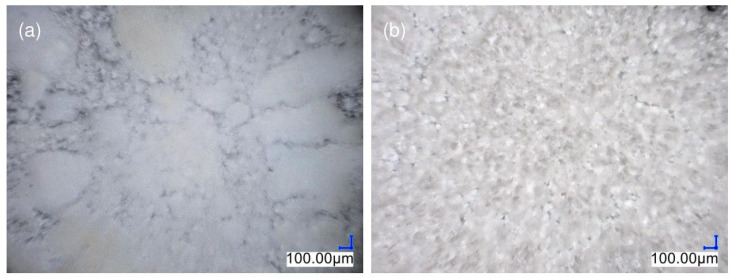
Optical microscopic images of (**a**) Aerosil and (**b**) Aerosil with modifier.

A total of 30 g of Aerosil200 and 30 g of an organosilicon modifier were weighed in a ceramic vessel with alumina balls. In the next step, the system was placed on rollers and mixing was continued for 24 h. The ABS copolymer, after pre-drying in an oven for 2 h at 70 °C, was loaded into a V-mixer, and then a pre-weighed silica modifier mixture (5% Aerosil 200, 5% modifier, 90% ABS) was added. Each of the systems was mixed in a mixer for 20 min. Microscopic images for all Aerosil + modifier mixtures looked analogous and are presented in Figure 1.

### 2.5. Injection Molding

**Figure 2:**
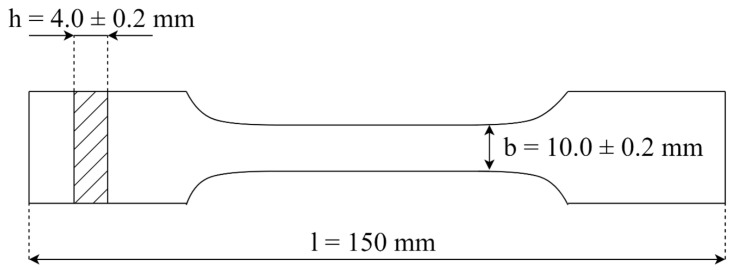
A 1A fitting with dimensions as follows: total length, l = 150 mm; thickness, h = 4 ± 0.2 mm; width of the measuring part, b = 10.0 ± 0.2 mm [33].

The samples were homogenized through the injection molding process. An Engel E-victory 170/80 tie-bar-less injection molding machine produced the test samples. The parameters of the injection process are presented in Table 2. Standardized type 1A fittings were obtained according to the [32] standard (Figure 2) for further testing.

### 2.6. Preparation of Filament

The injection samples were milled. The obtained granulate was homogenized during the extrusion of the stream with the HAAKE Rheomex OS.

The filament obtained in the previous stage was ground and extruded into a filament for 3D printing. Appropriate amounts of granules containing 5% modifier and ABS were weighed on a laboratory balance, and each system was diluted into six concentrations as follows: 0.1%, 0.25%, 0.5%, 1%, 1.5%, and 2.5%. The filament was extruded on a Filabot EX6 single-screw extruder with four heating zones (Table 3), an L/D 24 screw, and a nozzle with a diameter of 1.75 mm.

### 2.7. 3D Printing (FDM)

**Figure 3:**
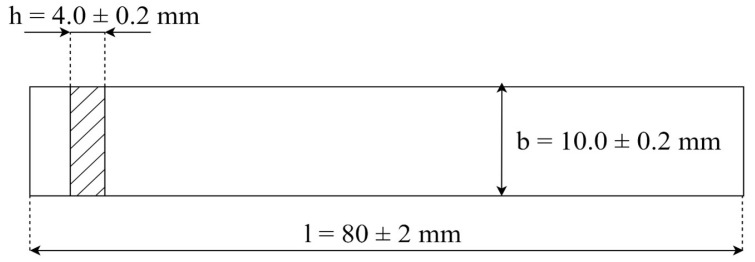
Dimensions of samples used for the bending test as follows: total length, l = 80 ± 2 mm; thickness, h = 4.0 ± 0.2 mm; width of the measuring part b = 10.0 ± 0.2 mm [33].

**Figure 4:**
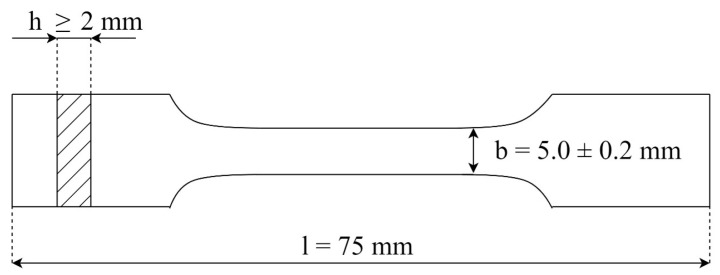
Dimensions of samples used for stretching test: total length l = 75 mm, thickness h ≥ 2 mm, the width of the measuring part, b = 5.0 ± 0.2 mm [33].

An extruded filament was used for FDM printing. The samples were printed on a Flashforge Guider IIs with a closed working chamber, a heated bed, and an extruder heating up to a maximum temperature of 300 °C. Parameters of printing are collected together and shown in Table 4. Bars for impact and bending tests were printed (Figure 3) as were paddles for stretching tests (Figure 4).

## 3. Results and Discussion

### 3.1. Chemical Characterization of Modifiers

**Figure 5:**
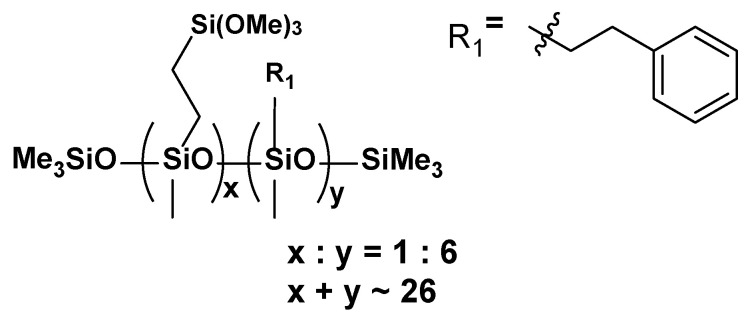
The formula of VT:6STYR.

**Figure 6:**
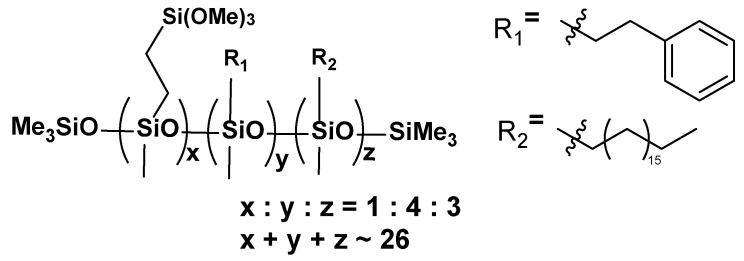
The formula of VT:4STYR:2OD.

**Figure 7:**
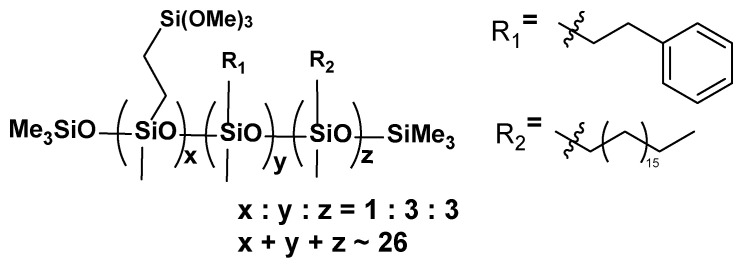
The formula of VT:3STYR:3OD.

**Figure 8:**
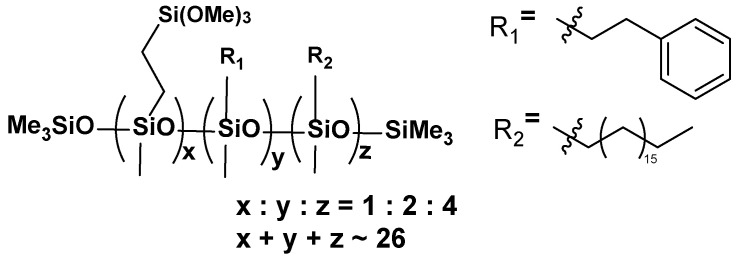
The formula of VT:2STYR:4OD.

**Figure 9:**
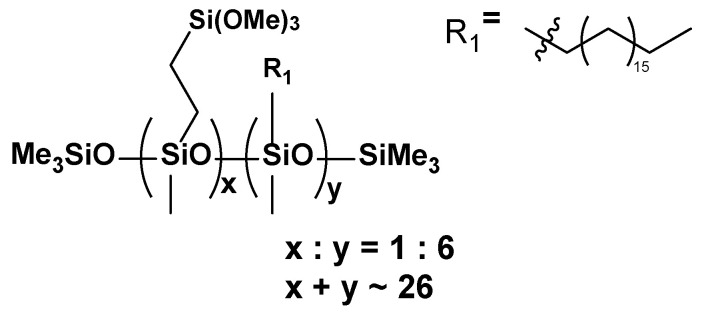
Formula of VT:6OD.

The hydrosilylation process was carried out until the disappearance of the signal from the Si–H group (the strong band from stretching vibrations at 2140 cm^−1^ and the bending vibrations at the wavelength of 888 cm^−1^), which indicates the complete conversion of polysiloxane. All compounds were obtained at a high yield (>94%) (Figure 5, Figure 6, Figure 7, Figure 8 and Figure 9). To validate compound structure, purity, and conversion, NMR analysis (^1^H, ^13^C, ^29^Si) was performed. The following signals were assigned:

**VT:6STYR**

**^1^H NMR** (400 MHz, CDCl_3_): σ(ppm) = 7.44–7.16 (m, Ph), 6.78, 6.75, 6.74, 6.71, 5.80, 5.75, 5.28, 5.25, 3.55 (s, -Si(OCH_3_)_3_), 2.71 (m, Ph-CH_2_CH_2_-Si-), 2.62, 2.38 (solvent), 2.18, 1.52, 1.41, 1.15, 0.94 (m, Ph-CH_2_-CH_2_-Si), 0.86, 0.64 (m, -SiCH_2_CH_2_Si-), 0.57, 0.15, 0.07 (-Si(CH_3_)_3_, -SiCH_3_)

**^13^C NMR** (101 MHz, CDCl_3_): σ(ppm) = 144.61, 144.33, 144.15, 137.98, 137.69, 137.01, 129.17 (solvent), 128.63–124.88 (m, Ph), 113.90, 50.63, 30.86, 30.72, 29.27, 29.15, 29.02, 21.59, 19.73, 19.52 (-CH_2_-Ph), 15.76, 15.01 (-Si-CH_2_), 14.80, 8.50, 2.05, −0.17, −1.97 (m, -Si(CH_3_)_3_, -SiCH_3_)

**^29^Si NMR** (79.5 MHz, CDCl_3_): σ(ppm) = −22.68–(−23.01) (SiMe, SiMe_3_), −41.96 (SiOMe_3_

**VT:4STYR:2OD**

**^1^H NMR** (400 MHz, CDCl_3_): σ(ppm) = 7.19, 7.13 (m, Ph), 3.53 (s, -Si(OCH_3_)_3_), 2.67 (m, Ph-CH_2_CH_2_-Si-), 2.36 (solvent), 1.55–1.26 (Si-CH_2_(CH_2_)_16_CH_3_), 0.88 (t, -CH_2_-CH_3_), 0.52 (-SiCH_2_(CH_2_)_16_CH_3_), 0.08 (-Si(CH_3_)_3_, -SiCH_3_)

**^13^C NMR** (101 MHz, CDCl_3_): σ(ppm) = 129.18 (solvent), 128.37–127.81 (m, Ph), 32.09, 29.89, 29.53 (-CH_2_-), 22.85 (-CH_3_), 14.27 (-Si-CH_2_), 0.12 (-Si(CH_3_)_3_, -SiCH_3_)

**^29^Si NMR** (79.5 MHz, CDCl_3_): σ(ppm) = −22.08–(−23.53) (SiMe, SiMe_3_), −41.92 (SiOMe_3_)

**VT:3STYR:3OD**

**^1^H NMR** (400 MHz, CDCl_3_): σ(ppm) = 7.14 (m, Ph), 3.53 (s, -Si(OCH_3_)_3_), 2.66 (m, Ph-CH_2_CH_2_-Si-), 2.36 (solvent), 1.54–1.25 (Si-CH_2_(CH_2_)_16_CH_3_), 0.88 (t, -CH_2_-CH_3_), 0.51 (-SiCH_2_(CH_2_)_16_CH_3_), 0.08–0.07 (-Si(CH_3_)_3_, -SiCH_3_)

**^13^C NMR** (101 MHz, CDCl_3_): σ(ppm) = 129.18 (solvent), 128.37–127.81 (m, Ph), 32.09, 29.90, 29.53 (-CH_2_-), 22.85 (-CH_3_), 14.27 (-Si-CH_2_)

**^29^Si NMR** (79.5 MHz, CDCl_3_): σ(ppm) = −22.53–(−24.08) (SiMe, SiMe_3_), −41.78 (SiOMe_3_)

**VT:2STYR:4OD**

**^1^H NMR** (400 MHz, CDCl_3_): σ(ppm) = 7.18 (solvent), 3.55 (s, -Si(OCH_3_)_3_), 2.36 (solvent), 1.54, 1.25 (m, Si-CH_2_(CH_2_)_16_CH_3_), 0.88–0.86 (t, -CH_2_-CH_3_), 0.51 (m, -SiCH_2_CH_2_Si-), 0.07 (m, -Si(CH_3_)_3_, -SiCH_3_)

**^13^C NMR** (101 MHz, CDCl_3_): σ(ppm) = 128.37–127.81 (m, Ph), 32.10, 29.91, 29.54 (-CH_2_-), 22.85 (-CH_3_), 14.27 (-Si-CH_2_)

**^29^Si NMR** (79.5 MHz, CDCl_3_): σ(ppm) = −22.49–(−23.94) (SiMe, SiMe_3_), −41.59 (SiOMe_3_)

**VT:6OD**

**^1^H NMR** (400 MHz, CDCl_3_): σ(ppm) = 7.18 (solvent) 3.55 (s, -Si(OCH_3_)_3_), 2.36 (solvent), 1.55, 1.26 (m, Si-CH_2_(CH2)_16_CH_3_), 0.88 (t, -CH_2_-CH_3_), 0.50 (m, -SiCH_2_CH_2_Si-), 0.04 (m, -Si(CH_3_)_3_, -SiCH_3_)

**^13^C NMR** (101 MHz, CDCl_3_): σ(ppm) = 129.18 (solvent), 32.11, 29.93, 29.86, 29.56 (-CH_2_-), 22.86 (-CH_3_), 14.27 (-Si-CH_2_)

**^29^Si NMR** (79.5 MHz, CDCl_3_): σ(ppm) = −22.06–(−23.01) (SiMe, SiMe_3_), −41.78 (SiOMe_3_)

### 3.2. Thermal Analysis (TGA and DSC)

Thermogravimetric analysis (TGA) and differential scanning calorimetry (DSC) were performed for both reference and ABS with unmodified SiO_2_ as well as ABS/SiO_2_/organosilicon modifier composites.

#### 3.2.1. Thermogravimetric Analysis (TGA)

**Figure 10:**
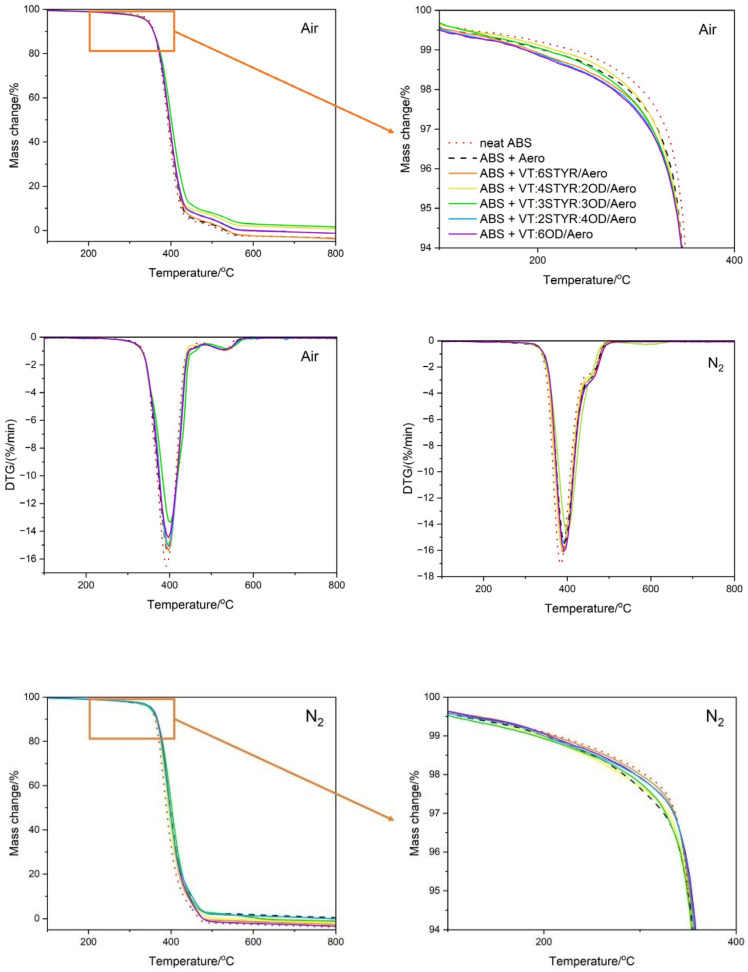
TGA and DTG curves of the ABS/SiO_2_/modifier composite in air and nitrogen atmospheres.

The determined parameters, including temperatures of 1% and 5% mass loss, the temperature of the start of degradation, and the temperature of the maximum rate of mass loss, are summarized in Table 5 and Table 6. The process of the thermal decomposition of the samples was carried out in air and nitrogen atmospheres (Figure 10).

The study of thermal stability in an air atmosphere shows that the temperature of the onset of degradation is highest for the system that has the same molar ratio of styrene and octadecyl substituents (ABS + VT:3STYR:3OD/Aero); an excess within any of these groups causes a decrease in this temperature.

The thermal stability study in an inert gas atmosphere proved that the degradation onset temperature is utmost for the system that has the same molar ratio of styrene and octadecyl substituents (ABS + VT:3STYR:3OD/Aero); an excess of any of these groups causes a decrease in onset temperature. The temperature of the onset of degradation in the nitrogen atmosphere is the lowest for the reference sample of neat ABS, and the modification further increases the thermal stability by 8.4 °C. The highest temperature at the maximum rate of mass loss occurs for the ABS + VT:3STYR:3OD/Aero composite. The difference between ABS + VT:3STYR:3OD/Aero and neat ABS is 15.8 °C. A loss of 1% of mass occurs latest (at the highest temperature) for a neat ABS sample. On the other hand, the temperature at 5% mass loss oscillates around 350 °C for both the reference samples and the modified ABS.

The temperature at the maximum weight loss is higher for modified ABS (in nitrogen and air) due to the higher Si−O bond energy (488 kJ/mol) in silica and the modifier than that of the C−C bonds (348 kJ/mol) and the C−H bonds (415 kJ/mol) in ABS copolymer.

#### 3.2.2. Differential Scanning Calorimetry (DSC)

**Figure 11:**
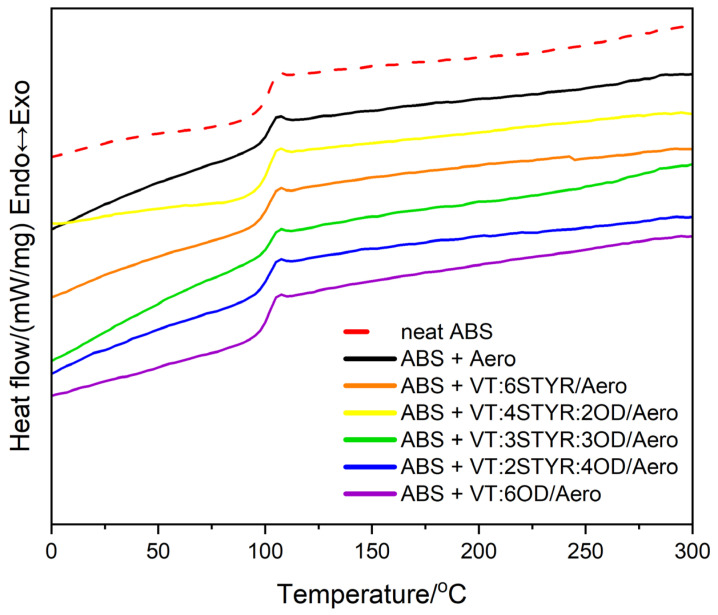
Results of the DSC analysis of neat ABS and itscomposites.

DSC analysis was performed to determine the effect of additives on phase transition temperatures such as the glass transition temperature (T_g_) of the ABS samples. Figure 11 shows the thermograms of pure ABS and ABS with the addition of 0.1% by weight of Aerosil 200 as well as with the addition of 0.1% of both silica and the organosilicon modifier.

The transition at a temperature of about 106 °C, which is visible in the thermographs, corresponds to the glass transition temperature (T_g_) that belongs to the second-order transformations. It is interpreted as a transition from a glassy state to a liquid state and is associated with the transition from the glassy phase to the rubber phase in this case. The addition of nanosilica and organosilicon modifiers does not significantly affect the change of the glass transition temperature of pure ABS (105.8 °C). The highest glass transition temperature is observed for ABS + 0.1% VT:2STYR:4OD/Aero (106.2 °C). The elevated T_g_ value following the introduction of the filler into the matrix is associated with the constrained thermal mobility of acrylonitrile butadiene styrene (ABS) chain segments. This phenomenon significantly influences the polymer’s processability by inducing a rise in system viscosity (see Section 3.4) [26]. ABS is an amorphous polymer, so no visible melting point is observed for the thermogram.

### 3.3. Testing the Composition of the Sample during Temperature Decomposition

**Figure 12:**
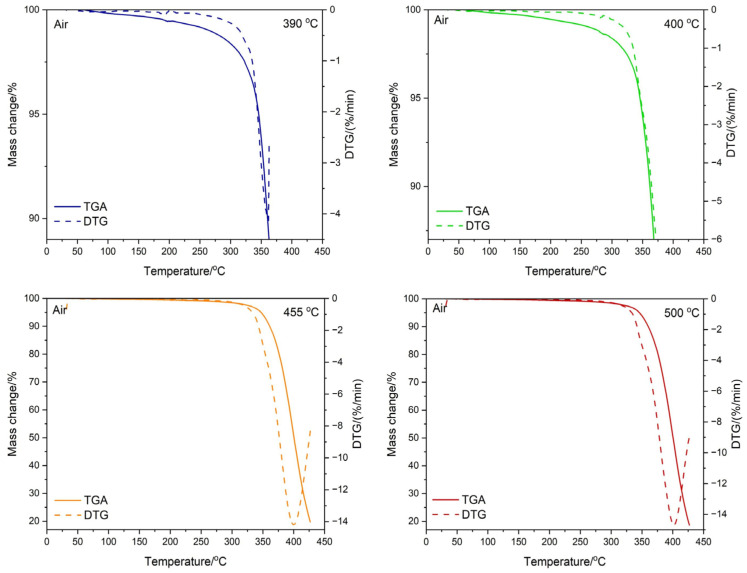
Results of thermogravimetric analysis for the ABS/VT:4STYR:2OD/Aero sample in an air atmosphere.

**Figure 13:**
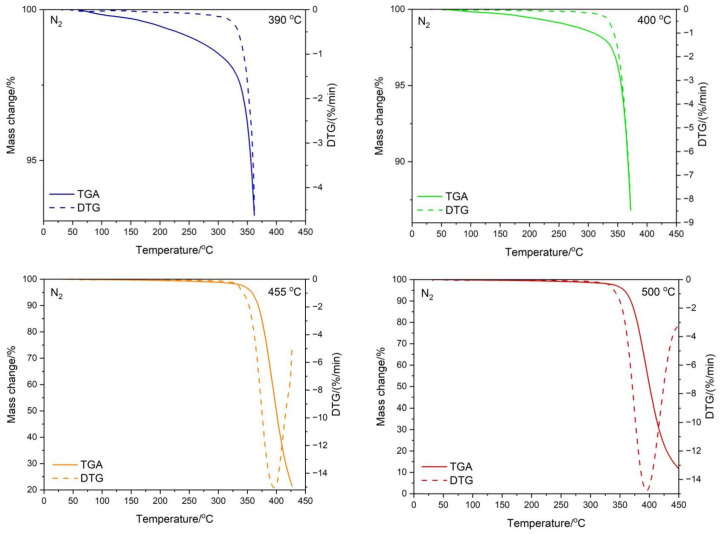
Results of thermogravimetric analysis for the ABS/VT:4STYR:2OD/Aero sample in a nitrogen atmosphere.

Thermogravimetric tests were carried out both in nitrogen and in the air for the ABS + VT:4STYR:2OD/Aero samples, which were carried out within various temperature ranges, i.e., from 30 °C to 390 °C, from 30 °C to 400 °C, from 30 °C to 455 °C, and from 30 °C to 500 °C (Figure 12 and Figure 13). The percentage of residual mass is summarized in Table 7.

**Figure 14:**
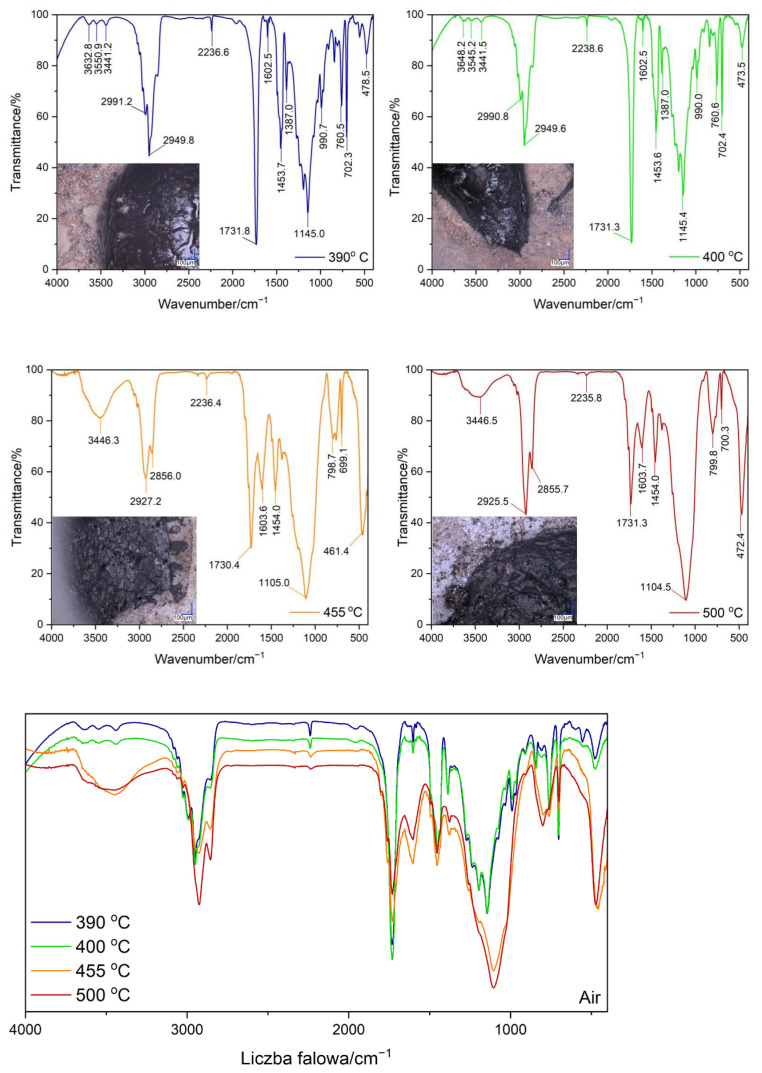
Results of FT-IR analysis with optical microscopic images of samples after TGA measurements in air atmosphere.

**Figure 15:**
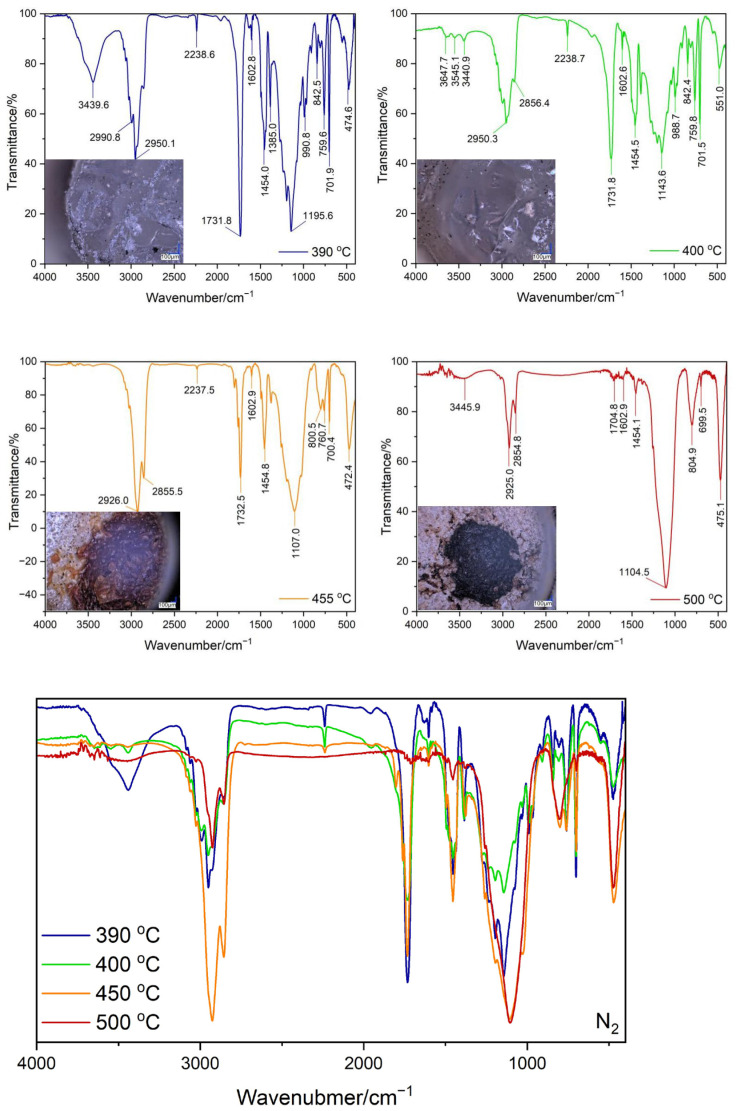
Results of FT-IR analysis with optical microscopic images of samples after TGA testing in a nitrogen atmosphere.

The images of the samples after the TGA test were made using an optical microscope at 100 and 200 times magnification, and the results are shown in Figure 13 and Figure 14. Transmission measurements were carried out for samples subjected to thermogravimetric analysis within various temperature ranges. Sample weights alongside KBr were ground in an agate mortar, and then pellets were made using a hydraulic press. The spectra of the samples, together with their microscopic images, are shown in Figure 14 (TGA in the air) and Figure 15 (TGA in nitrogen).

The broad peak near the value of 3440 cm^−1^ corresponds to the O−H stretching vibrations found in silica. As the degradation temperature increases, Si-O-Si stretching (about 1100 cm^−1^ and 800 cm^−1^) and the bending (460/470 cm^−1^) vibrations of nanosilica increase in intensity. This means that the mass percentage of Aerosil increases with the loss of volatile decomposition observed among products of the sample at the given temperatures. As the temperature increases, signals from vibrations in the aromatic ring, mainly that from C-H stretching vibrations near 3025 cm^−1^ originating in styrene, disappear in both ABS and the modifier. For a peak near 2235 cm^−1^ coming from the stretching vibrations of the carbon–nitrogen triple bond present in the polymer chain, complete disappearance is observed at 500 °C in a nitrogen atmosphere. Peak 1730 cm^−1^ corresponds to the stretching vibrations of the C−O double bond present in the decomposition products.

### 3.4. Rheology

**Figure 16:**
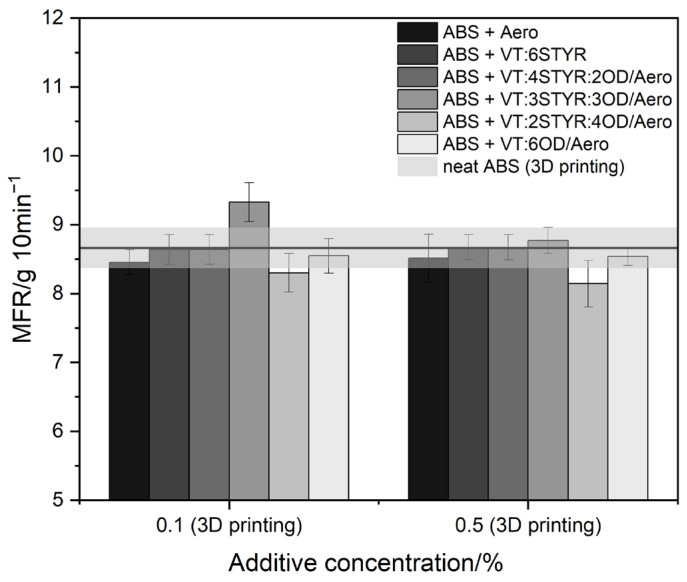
Result of MFR measurements.

Neat ABS’s melt flow rate (MFR) at 220 °C is 8.664 g/10 min. The addition of small amounts of modifiers does not significantly change the MFR value (Figure 16). The highest value of the MFR index is characterized by samples containing VT:3Styr:3OD/Aero with a concentration of 0.1%. The styryl groups are responsible for interactions with the ABS matrix through weak π-stacking interactions, while the octadecyl group causes a better plasticization effect of the system; therefore, they slightly improve the melt flow ratio. The addition of modifiers also prevents a decrease in the rheological parameters regardless of the amount of filler (Aero) added, which is important from the perspective of plastic processing.

### 3.5. Microscopy

#### 3.5.1. Optical Microscopy

**Figure 17:**
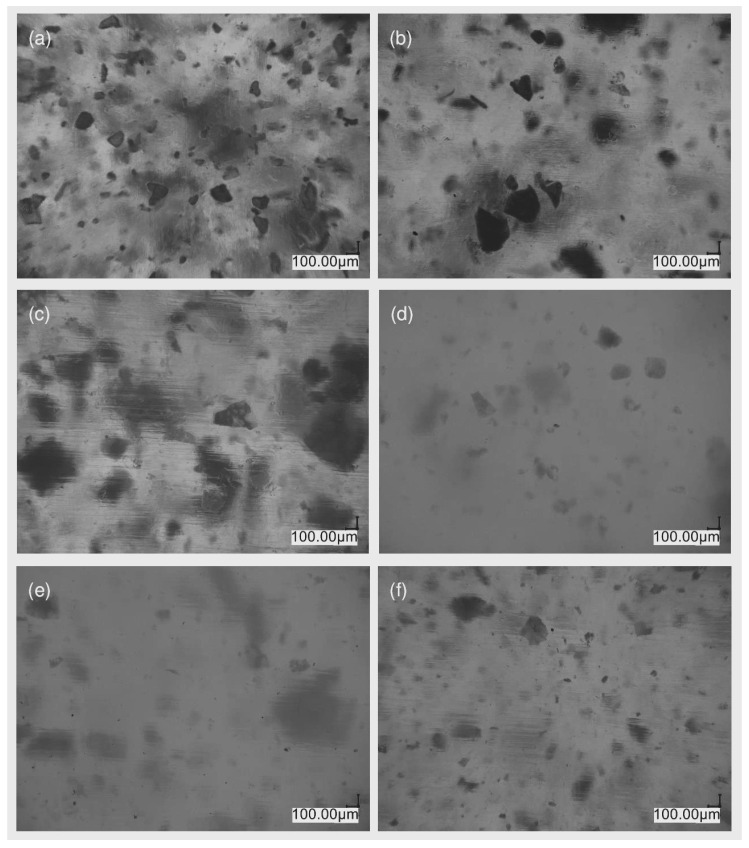
Optical microscopic images of injected ABS samples containing 5% by mass of (**a**) Aero, (**b**) VT:6STYR/Aero, (**c**) VT:4STYR:2OD/Aero, (**d**) VT:3STYR:3OD/Aero, (**e**) VT:2STYR:4OD/Aero, and (**f**) VT:6OD/Aero.

**Figure 18:**
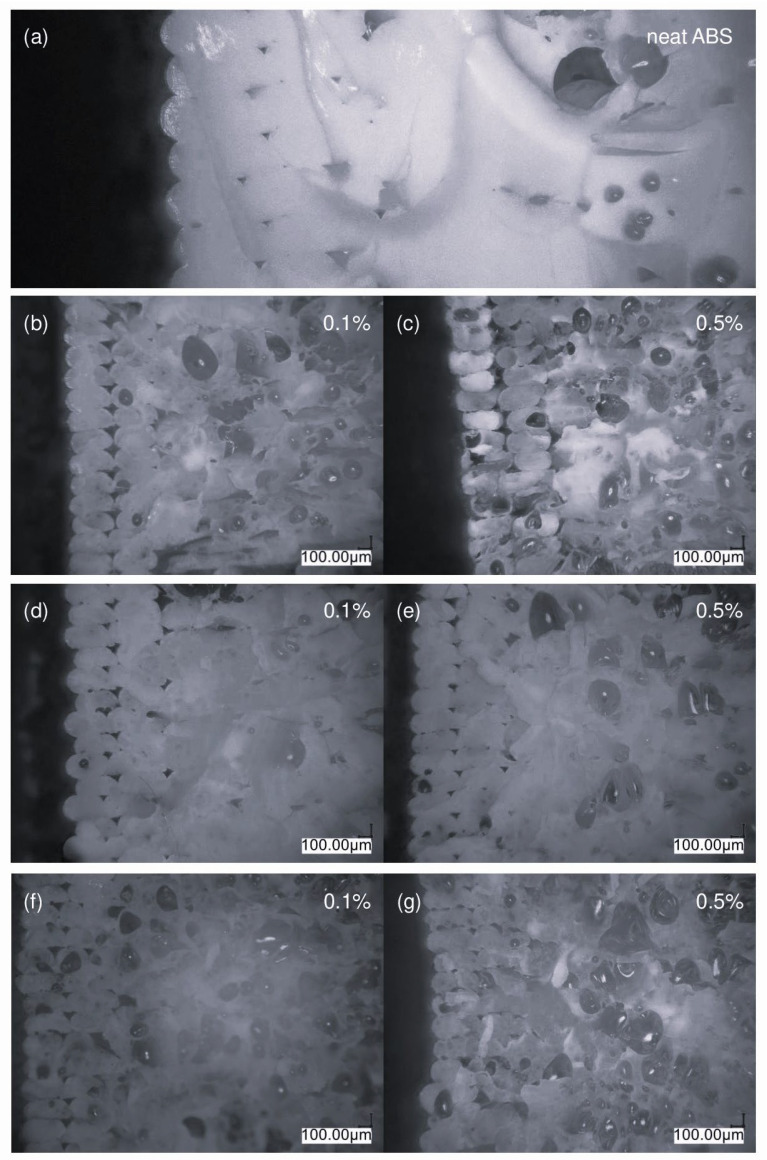

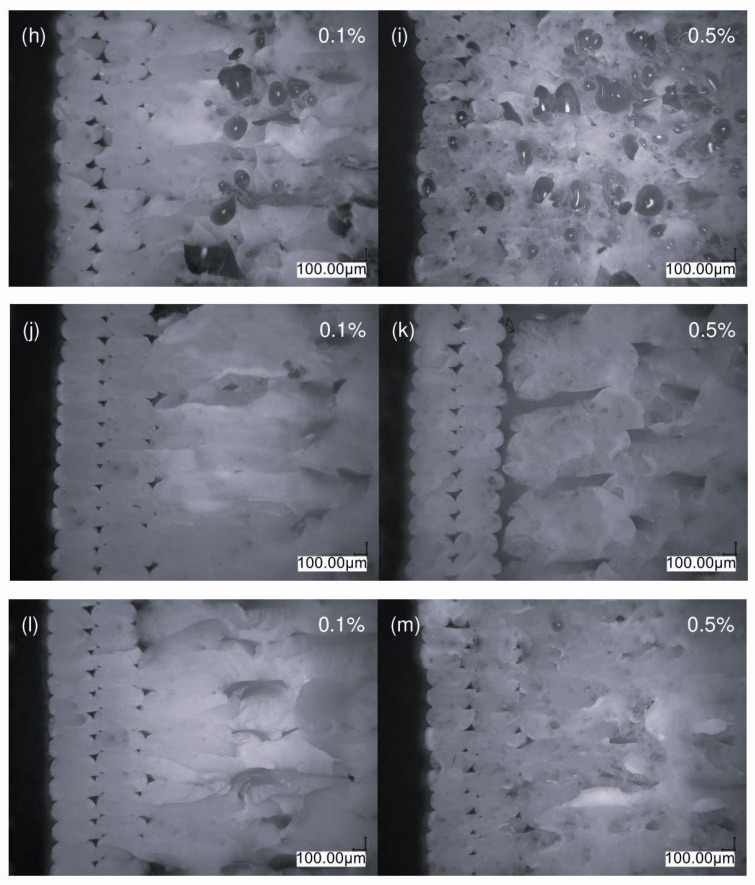
Optical microscopic images of the cross-sections of printed ABS samples after impact testing of (**a**) neat ABS and two selected concentrations as follows: (**b**,**c**) ABS/Aero, (**d**,**e**) VT:6STYR/Aero, (**f**,**g**) VT:4STYR:2OD/Aero, (**h**,**i**) VT:3STYR:3OD/Aero, (**j**,**k**) VT:2STYR:4OD/Aero, (**l**,**m**) VT:6OD/Aero.

Figure 17 and Figure 18 illustrate the structure of the injected and printed samples, respectively. In the case of samples obtained through injection molding, thanks to their transparency inside the polymer matrix, it is possible to observe the agglomerated particles of nanosilica in the photos.

The microscopic image of the reference sample (Figure 17a) shows nanosilica agglomerates present in the composite after the first homogenization step. Imaging was performed to pre-evaluate the dispersion of additives in the matrix prior to the extruder blending process. The addition of the modifier changed the degree of silica dispersion in the polymer matrix. The largest silica agglomerates are shown in image (Figure 17c), which presents a system containing the VT:4STYR:OD modifier. As the content of the OD groups in the modifier increases in subsequent samples (Figure 17d–f), there is improved dispersion of the additive in the polymer matrix.

Figure 18 shows the fractures of FDM-printed samples after being subjected to Charpy impact testing. At a higher concentration, more protrusions are visible between and within the layers. Neat ABS fractures exhibit a highly compact structure, with a significant interlayer contact area and minimal free spaces (Figure 18a).

The fracture of samples with a concentration of 0.1% additives has a more compact structure and more homogeneity than samples with a concentration of 0.5%, which is caused by a lower concentration of nanosilica. The inclusion of modified silica lessens the adhesive force between the layers of the substance. The ABS + VT:4STYR:2OD/Aero system (Figure 18f,g) features a less compact structure and numerous voids for both concentrations. It was discovered that the additive did not disperse well in the polymer matrix, as evidenced by the lack of homogeneity after the initial step of mixing (Figure 18c).

With the increase in the content of the OD groups in the modifier, cohesion between the layers is higher, and smaller free spaces are observed due to the additional OD groups having a plasticizing effect (Figure 18h–m). ABS + VT:6OD/Aero samples have the most solid structures, a relatively large contact area between the material layers, and small free spaces (Figure 18l,m).

#### 3.5.2. SEM-EDS

**Figure 19:**
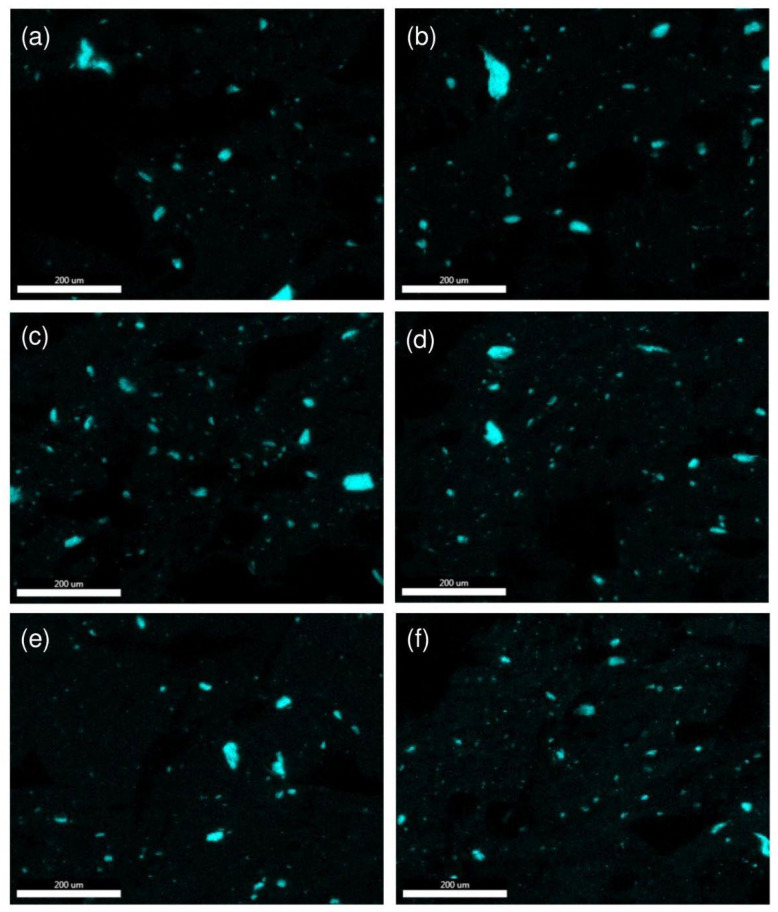
SEM with Si mapping (EDS) for samples of (**a**) ABS + 2.5% Aero, (**b**) ABS + 2.5% (VT:6STYR/Aero), (**c**) ABS + 2.5% (VT:4STYR:2OD/Aero), (**d**) ABS + 2.5% (VT:3STYR:3OD/Aero), (**e**) ABS + 2.5% (VT:2STYR:4OD/Aero), and (**f**) ABS + 2.5% (VT:6OD/Aero).

SEM-EDS microscopy was used to examine dispersion of filler in the matrix, and identify any agglomerates present. Figure 19. shows the mapping of silicon atoms in composites. Mapping was performed both for ABS with the addition of only nanosilica and for systems containing the following modifiers: VT:6STYR/Aero, VT:4STYR:2OD/Aero, VT:3STYR:3OD/Aero, VT:2STYR:4OD/Aero, and VT:6OD/Aero. Microscopic images were taken from the section of the injection-molded bar. All the systems are characterized by the presence of larger agglomerates, both for the reference sample without the addition of the modifier and for the modified samples. The SEM-EDS photos validate the observations made through optical microscopy. Specifically, these observations highlight that a higher content of alkyl groups, coupled with a lower content of styryl groups in the organosilicon modifiers, results in better filler dispersion.

### 3.6. Contact Angle Measurements

**Figure 20:**
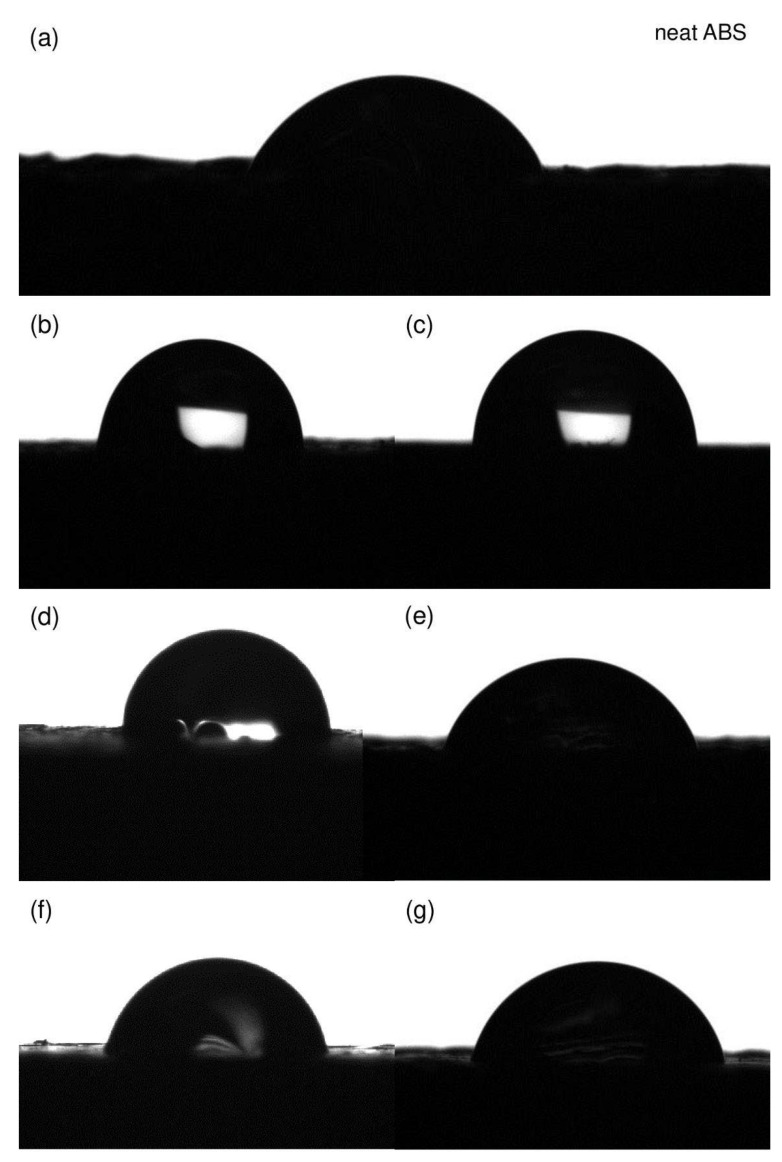
The water contact angle for samples of (**a**) neat ABS, (**b**) ABS + 2.5% Aero, (**c**) ABS + 2.5% (VT:6STYR/Aero), (**d**) ABS + 2.5% (VT:4STYR:2OD/Aero), (**e**) ABS + 2.5% (VT:3STYR:3OD/Aero), (**f**) ABS + 2.5% (VT:2STYR:4OD/Aero), and (**g**) ABS + 2.5% (VT:6OD/Aero).

Measurements of the water contact angle (WCA) were performed for the modifier/Aero/ABS composites obtained through the FDM method (Figure 20). Additionally, measurements were also carried out for the reference samples, i.e., those of neat ABS and ABS with unmodified silica. The contact angle of neat ABS was 70.9°, indicating the hydrophilic nature of the copolymer surface. The addition of silica changed the microstructure (increased roughness) of the composite, which resulted in achieving higher values of the contact angle. The contact angle analysis allows for the evaluation of the potential functional characteristics of novel materials with regard to their hydrophilic–hydrophobic properties.

For ABS samples modified by VT:6STYR/Aero, a significant increase in the contact angle is observed. For 2.5% of the modifier concentration, the contact angle reached a value above 90°, which proves the hydrophobic properties of the material. High contact angle values were also achieved for VT:4STYR:2OD (92.9° for 2.5% concentration). VT:6STYR and VT:4STYR:2OD modifiers have phenylethylene groups in their structure, which are compatible with the polymer matrix. Additionally, OD consists of long alkyl chains that are responsible for hydrophobic properties.

Adding other organosilicon modifiers to ABS + Aero systems results in forming a hydrophilic surface for most systems (Table 8).

Based on the analysis of microscopic images and contact angle values, a correlation can be observed. Neat ABS exhibits hydrophilic properties and has a compact and uniform internal structure (as shown in Figure 18). This results in a relatively flat surface when printed. However, the addition of Aerosil causes the surface to become more irregular with the formation of cracks and large protrusions. The degree of irregularity is higher at 0.5% compared with 0.1%, resulting in a more hydrophobic surface with a larger water contact angle. ABS + VT:6OD/Aero displays the most homogeneous cross-section and a hydrophilic surface. For the other systems, the impact of the modification on surface properties is minimal, which is likely due to the formation of modified silica agglomerates.

### 3.7. Mechanical Properties

A summary of the results of mechanical tests carried out on modified samples obtained through both 3D printing and traditional injection molding will be discussed.

#### 3.7.1. Tensile Strength

Printed samples with a concentration of between 0.1% and 0.5% additives as well as injected samples with a concentration of 5% additives were subjected to a tensile strength test.

**Figure 21:**
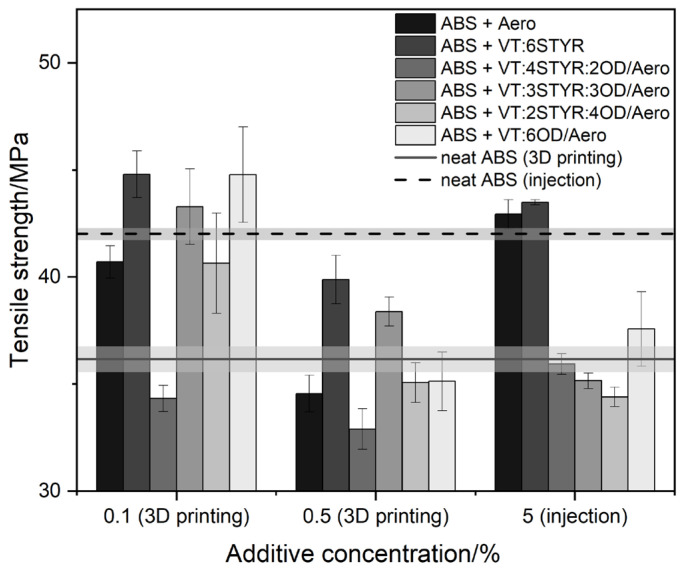
Tensile strength of ABS/modifier/Aero in 3D printing and injection molding.

The tensile strength values for neat ABS were 36.4 ± 1.1 MPa for samples obtained through FDM printing and 42.0 ± 0.3 MPa for samples obtained through injection molding (Figure 21). The higher value of tensile strength of a neat ABS sample obtained by injection molding than that by FDM method results from the specificity of a given process and the related more compact structure of the injected samples. Similar results were obtained by Dawoud et al. in their earlier research [34]. The tensile stress values of injected samples are highest for Aero/ABS and VT:6Styr/Aero/ABS; the addition of other modifiers reduces the stress value.

The tensile stress values of 3D printed samples are highest for systems containing organosilicon modifiers. In addition, at a lower modifier concentration of 0.1%, these values exceed the values of neat ABS samples obtained through injection. Even a small amount of the additive improves the mechanical properties of composites, which are beneficial in economic terms.

The highest tensile strength values among printed composites are observed among systems with a concentration of 0.1% as follows: VT:6STYR/Aero, VT:3STYR:3OD/Aero, and VT:6OD/Aero. Due to the compatibility of the phenylethylene groups of the modifier (VT:6STYR/Aero) with the ABS chain, the strength of the material is higher. The increase in the tensile strength parameters of the composite containing the largest number of the OD groups is due to the increased plasticizing effect that resulted in a greater level of homogeneity within the internal structure of the sample. The VT:3STYR:3OD/Aero composite has an indirect influence on both groups and, hence, also has high strength values.

At higher concentrations, due to a higher proportion of silica, as shown in microscopic images of cross-sections, it causes greater heterogeneity in the structure of composites, which results in a decrease in their tensile strength.

#### 3.7.2. Elongation

**Figure 22:**
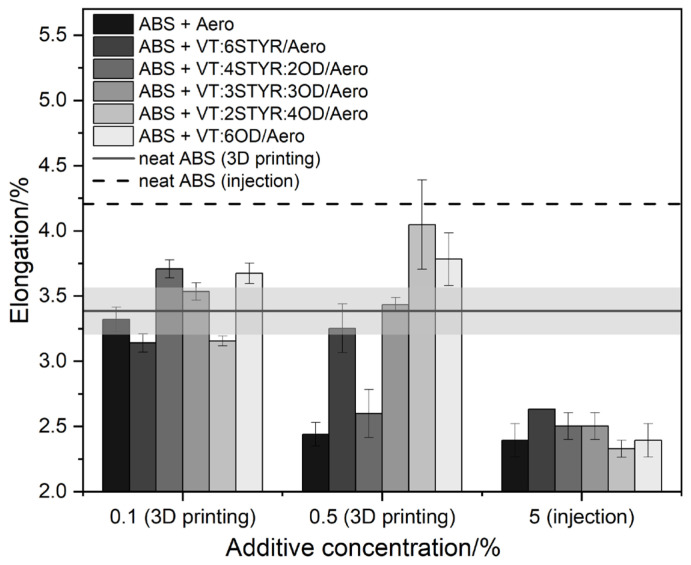
Elongation at maximum load for ABS/modifier/Aero in 3D printing and injection molding.

According to Figure 22, elongation at the maximum load for neat ABS samples is 3.39% and 4.21% for 3D printed and injection molded samples, respectively. The injection molded samples with silica show lower elongation values due to increased brittleness and silica agglomeration in the material matrix. Silica is added to plastics in order to increase the strength and hardness of materials because it acts as a filler that increases resistance to abrasion and mechanical damage. Values of tensile strength (See Section 3.7.1) and hardness (See Section 3.7.4) are higher for composites compared with neat ABS, which is associated with lower flexibility. However, the effect of silica in printed samples is limited, and the modifier has a greater influence on elongation values that are around the reference sample value. In samples VT:2STYR:4OD and VT:6OD with a concentration of 0.5%, the elongation value is higher due to the plasticizing effect of the OD groups, resulting in increased flexibility.

#### 3.7.3. Young’s Modulus

**Figure 23:**
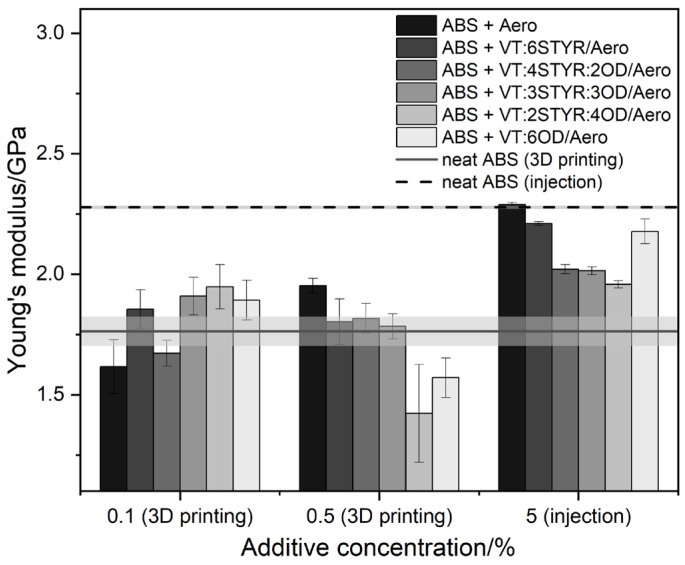
Tensile modulus of ABS samples in 3D printing and injection molding.

The addition of modifiers or filler has no significant impact on Young’s modulus. The modulus of linear deformation (Figure 23) reaches the highest values for samples obtained through injection molding. The addition of modifiers slightly reduces the module value. In the case of samples obtained through the FDM technique, a slight increase in the modulus value is observed at the lowest concentration of 0.1%.

#### 3.7.4. Impact Test and Hardness

**Figure 24:**
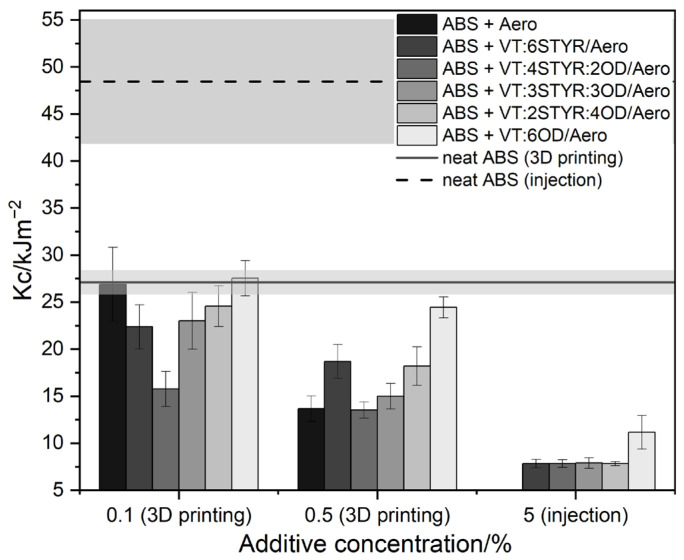
Impact strength of ABS samples in 3D printing and injection molding.

The impact strength of 3D printed neat ABS is 27.1 ± 1.3 [kJ/m^2^]. However, during testing, most systems showed a lower level of fracture toughness compared with the neat ABS reference sample (Figure 24). This can be attributed to the lower homogeneity and cohesion of the printed samples containing nanosilica, as seen in the cross-sectional images (Figure 18).

The presence of silica as a filler increases the hardness and stiffness of plastics, resulting in a loss of flexibility and lower impact strength. However, the addition of modifiers containing 6OD and 2STYR:4OD improves the impact strength of samples with Aerosil. The lowest concentration of 0.1% VT:6OD/Aero even shows values above the neat ABS range.

The impact strength of neat ABS obtained through injection molding is 48.4 ± 6.6 [kJ/m^2^]. The addition of nanosilica to ABS samples results in a deterioration in fracture toughness. This is due to the formation of agglomerates in systems above 3 wt%, as shown in the optical microscopic images (Figure 17).

Shore hardness tests were conducted to confirm the reinforcing effect of silica. The Shore D hardness (Table 9) of printed neat ABS is 59. The obtained composites have higher hardness compared with neat ABS, as expected. Silica has a high hardness, which reinforces the plastic to which it is added. The ABS + VT:3STYR:3OD/Aero system has the highest values.

## 4. Conclusions

The obtained results confirm the effect of the addition of modified silica with organosilicon compounds on the improvement of the mechanical properties of printed composites based on the ABS matrix. This is important in the case of FDM technology because 3D printed objects usually have much lower levels of resistance than injected ones do, which are related to different specificities used in the operation of both methods.

To the best of our knowledge, an endeavor has been undertaken to delineate the thermal decomposition process of ABS composites in this study for the first time. The mechanism of the thermal decomposition of the tested systems was determined using additional techniques (FT-IR, optical microscope). The presented results constitute an important contribution to research on the thermal stability of ABS.

Microscopic examination (digital microscope and SEM-EDS) has proven the positive effect of the addition of octadecyl groups on the increased dispersion of silica in composites. The best results were obtained with the lowest concentration of additives (0.1%). The properties of the composites can be notably influenced by the addition of a minimal quantity of additives, thus presenting economic advantages.

The compatibility of the introduced phenylethylene groups and the plasticizing effect of the OD groups affected both the tensile strength and the surface character of the composites. Silica in the composite increases the contact angle of the surface, making it hydrophobic due to structural irregularities. The modifier altered the contact angle value by smoothing the composite surface, thereby making it hydrophilic.

The obtained results indicate the potential benefits of using silica that is functionalized with organosilicon compounds as a modifier in 3D printing, introducing significant changes in both the surface, strength, and thermal properties of ABS composites. These findings may be used in further work on improving 3D printing technology and modifying composite materials.

## Figures and Tables

**Table 1:** Amounts of olefins used in the reactions.

Code	Amount of VT/g	Amount of STYR/g	Amount of OD/g
VT:6STYR	11.0	46.5	-
VT:4STYR:2OD	11.0	31.0	37.6
VT:3STYR:3OD	11.0	23.2	56.4
VT:2STYR:4OD	11.0	15.5	75.1
VT:6OD	11.0	-	112.6

**Table 2:** The parameters of the injection process.

Properties	Parameters
Maximum dispensing time	15.0 s
Dispensing volume	31.00 cm^3^
Holding time	7 s
Cooling time	35.00 s
Holding pressure	500.0–1100.0 bar
Mold temperature	70 °C
Dosing efficiency	0.71 cm^3^/s

**Table 3:** The parameters of the filament extrusion.

Properties	Parameters
Zone 1 temperature	215 °C
Zone 2 temperature	240 °C
Zone 3 temperature	230 °C
Filling zone temperature	90 °C
Voltage	20–25 V
Current	1–1.5 A

**Table 4:** The parameters used for 3D printing.

Properties	Parameters
Nozzle diameter	0.4 mm
Extruder temperature	225 °C
Bed temperature	105 °C
Layer height	0.2 mm
Bottom and top layer style	linear
Fill style	linear
Infill density	100%
Printing speed	60 mm/s

**Table 5:** Thermogravimetric analysis results in an air atmosphere.

Code	1% of Weight Loss/°C	5% of Weight Loss/°C	Onset Temperature/°C	Temperature at the Maximum Rate of Mass Loss/°C
neat ABS	235.7	346.6	363.7	392.6
ABS + 0.1%Aero	205.5	342.8	364.0	396.3
ABS + 0.1% VT:6STYR/Aero	191.9	341.2	365.6	397.1
ABS + 0.1% VT:4STYR:2OD/Aero	217.2	342.1	363.9	395.0
ABS + 0.1% VT:3STYR:3OD/Aero	207.8	341.0	366.9	399.7
ABS + 0.1% VT:2STYR:4OD/Aero	187.3	340.7	365.3	397.1
ABS + 0.1% VT:6OD/Aero	187.1	340.6	364.5	396.3

**Table 6:** Thermogravimetric analysis results in a nitrogen atmosphere.

Code	1% of Weight Loss/°C	5% of Weight Loss/°C	Onset Temperature/°C	Temperature at the Maximum Rate of Mass Loss/°C
neat ABS	212.9	350.2	361.9	384.3
ABS + 0.1%Aero	200.6	349.7	367.9	393.2
ABS + 0.1% VT:6STYR/Aero	209.6	351.3	365.1	389.7
ABS + 0.1% VT:4STYR:2OD/Aero	196.3	350.1	365.4	390.0
ABS + 0.1% VT:3STYR:3OD/Aero	191.3	349.1	370.3	400.1
ABS + 0.1% VT:2STYR:4OD/Aero	200.2	352.5	366.7	391.5
ABS + 0.1% VT:6OD/Aero	205.8	353.8	368.5	393.2

**Table 7:** Results of thermogravimetric analysis of the ABS + VT:4STYR:2OD/Aero sample at different temperatures.

	Residual Mass/°C	
Conditions	N_2_	Air
390 °C	92.75	88.86
400 °C	86.40	84.35
455 °C	21.27	18.60
500 °C	5.14	17.46

**Table 8:** Water contact angle.

Code	Contact Angle/°						
	Concentration of Additives/%						
	-	0.1	0.25	0.5	1	1.5	2.5
neat ABS	70.9 ± 3.2	-	-	-	-	-	-
ABS + Aero	-	86.2 ± 1.3	89.6 ±1.4	93.9 ± 2.0	89.8 ± 3.5	81.2 ± 0.9	91.7 ± 2.4
ABS + VT:6STYR/Aero	-	76.5 ± 0.5	76.6 ±0.6	74.9 ± 2.0	79.2 ± 0.5	86.4 ± 3.1	92.9 ± 2.0
ABS + VT:4STYR:2OD/Aero	-	90.5 ± 0.5	86.8 ± 1.2	87.4 ± 3.7	84.8 ± 3.9	85.0 ± 0.5	92.9 ± 1.9
ABS + VT:3STYR:3OD/Aero	-	86.6 ± 2.5	84.1 ± 2.1	88.6 ± 2.6	82.1 ± 0.8	73.0 ± 1.6	71.7 ± 1.2
ABS + VT:2STYR:4OD/Aero	-	85.3 ± 0.8	85.5 ± 3.2	83.2 ± 1.2	82.8 ± 1.4	72.1 ± 2.6	79.1 ± 3.3
ABS + VT:6OD/Aero	-	74.9 ±0.5	77.4 ± 1.8	76.6 ± 1.1	79.5 ± 2.6	77.2 ± 0.4	79.1 ± 2.6

**Table 9:** Hardness in Shore D scale.

Code	Shore Hardness						
	Concentration of Additives/%						
	-	0.1	0.25	0.5	1	1.5	2.5
neat ABS	59	-	-	-	-	-	-
ABS + Aero	-	71	66	71	71	66	63
ABS + VT:6STYR/Aero	-	68	66	72	73	66	68
ABS + VT:4STYR:2OD/Aero	-	72	74	79	74	73	74
ABS + VT:3STYR:3OD/Aero	-	75	77	79	78	77	79
ABS + VT:2STYR:4OD/Aero	-	76	69	75	76	72	78
ABS + VT:6OD/Aero	-	78	68	76	77	78	77

## Data Availability

Data are contained within the article.
